# Mental Health of Brazilians Heart Surgeons: Cross-Sectional Study

**DOI:** 10.21470/1678-9741-2020-0335

**Published:** 2021

**Authors:** Eduardo Augusto Victor Rocha, Fernanda Roquette de Araujo, Ana Carolina Madureira Nunes, Luiza Lins Khoury, Bárbara Stéphane de Macedo Guedes, Luana Albuquerque Pessoa, Débora Rodrigues Tolentino, Ana Carolina de Almeida Borges Santos, Gustavo Lembi Magalhães

**Affiliations:** 1 Department of Surgery, Faculdade de Saúde e Ecologia Humana (FASEH), Vespasiano Minas Gerais, Brazil.; 2 Faculdade de Saúde e Ecologia Humana (FASEH), Vespasiano, Minas Gerais, Brazil.; 3 Brazilian Society of Psychiatry (ABP), Betim, Minas Gerais, Brazil.

**Keywords:** Mental Health, Depression, Burnout, Psychological, Substance-Related Disorders, Surgeons

## Abstract

**Introduction:**

Over the past few years, we have seen some signs of change in mental health among cardiovascular surgeons. Suicide cases, troubled professional relationships, separations, and treatment of depression and anxiety are common occurrences in this group of surgeons. With this in mind, we decided to perform an analysis of the mental health of Brazilian cardiovascular surgeons.

**Methods:**

This is a cross-sectional qualitative study. Thirty-seven validated questionnaires (from the Diagnostic and Statistical Manual of Mental Disorders, fifth edition) were collected at the 46th Congress of the Sociedade Brasileira de Cirurgia Cardiovascular, in April 2019. It was authorized by the Faculdade da Saúde e Ecologia Humana Ethics Committee (CAAE-09479519.7.0000.5101). The questionnaires were analyzed by a psychiatrist who grouped the individuals with signs suggestive of some mental disorder.

**Results:**

The questions that pointed out signs and symptoms of possible anxiety, depression, alcohol or drug abuse, and burnout were selected in the questionnaire. Seventeen individuals (45.95%) did not score for any disorder. Twenty individuals (54.05%) in our sample had one or more disorders, with 43.24% (16 individuals) showing signs or symptoms compatible with anxiety - the World Health Organization data for Brazil show a 9.3% incidence of anxiety in the general population. We found signs of depression in 21.62% of our sample (5.8% in the general population), of alcohol or drug abuse in 27.03% (19.4% in the general population), and of burnout in 40.54% (32% in the general population).

**Conclusion:**

Mental disorders are present in most cardiovascular surgeons studied.

**Table:** 

Abbreviations, acronyms & symbols	
**DSM-5(R)**	**= Diagnostic and Statistical Manual of Mental** **Disorders, fifth edition**
**FICF**	**= Free and informed consent form**
**OR**	**= Odds ratio**
**SBCCV**	**= Sociedade Brasileira de Cirurgia Cardiovascular**

## INTRODUCTION

Knowing the finitude of life and facing the suffering of patients during the physicians’ professional practice make medical activity extremely arduous. The burnout syndrome can be defined as a state of exhaustion caused by professional activity^[1]^. Some studies demonstrate that about 35% of surgeons have burnout^[1]^. Because it deals with severe patients and risk situations, the cardiovascular surgery is one of the medical specialties that generates most impact on mental health. The cardiovascular surgery environment demands a lot of the physicians and many important decisions must be made based in rationality and emotional balance. Surgeons work hard for many hours, regularly dealing with life and death situations for their patients. They make many personal sacrifices to practice their profession. These attributes of surgical practice added to the rigors and duration of training can interfere with mental health. A surgeon's routine includes not having schedules and often not fulfilling social and family commitments. According to one study, the divorce rate among surgeons is one of the highest, reaching 33% after 30 years^[2]^. There is not a very reliable statistic, due to the lack of studies on the subject, but in the United States of America, an average of 10-15% of debts due to alcohol abuse among doctors is already calculated^[3]^. Rates of suicide among surgeons and physicians have been reported to be remarkably higher than in the general population^[4]^. Controlling emotions and personal problems is fundamental for better results at work. Suicide, divorces, and dysfunction in personal relationships motivated this study. There is a fine line that separates dedication and overwork on mental health^[1]^.

### Objectives

The objective of this study is to identify the prevalence of mental health of Brazilian cardiovascular surgeons, at the time of the 46^th^ Congress of the Sociedade Brasileira de Cirurgia Cardiovascular (SBCCV). With these results, we intend to contribute with the specialty in order to improve the training and work environment, besides stimulating research in this area.

## METHODS

We conducted a qualitative cross-sectional study by using a convenience sample and evaluating the mental health of cardiovascular surgeons present at the 46^th^ Brazilian congress of SBCCV, April 2019, in Belo Horizonte, Minas Gerais. We distributed 120 validated questionnaires from the Diagnostic and Statistical Manual of Mental Disorders, fifth edition (DSM-5(R)), by the American Psychiatric Association^[5]^. The questionnaires were distributed during the breaks of the congress and collected at the end of the event - those were deposited in a box anonymously by the participants at the congress secretariat. Thirty-seven questionnaires were received complete with signed free and informed consent form (FICF). The remaining questionnaires were not fully answered by the participants or the individuals did not want to participate in the study.

The inclusion criterion was being a graduated cardiovascular surgeon who is practicing in Brazil and answered the entire questionnaire.

The exclusion criteria were being medical students, residents, and other professionals who are not doctors or incomplete or unsigned forms.

All participants read and signed the FICF.

The questionnaire separates individuals into four nosological entities, namely: anxiety, depression, substance abuse, and burnout. Surgeons showing signs of one or more disorders were separated into single and mixed disorder groups. The results were analyzed blindly by a psychiatrist who grouped the individuals with signs suggestive of some mental disorder, among the four abovementioned.

The work was analyzed and approved by the Research Ethics Committee of the Faculdade da Saúde e Ecologia Humana, under the number CAAE-09479519.7.0000.5101.

## RESULTS

**Fig. 1:**
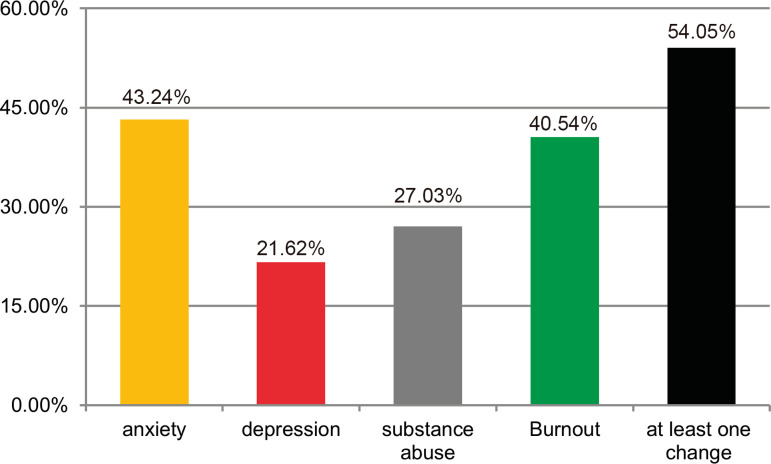
Prevalence of mental health disorders in the population studied.

**Fig. 2:**
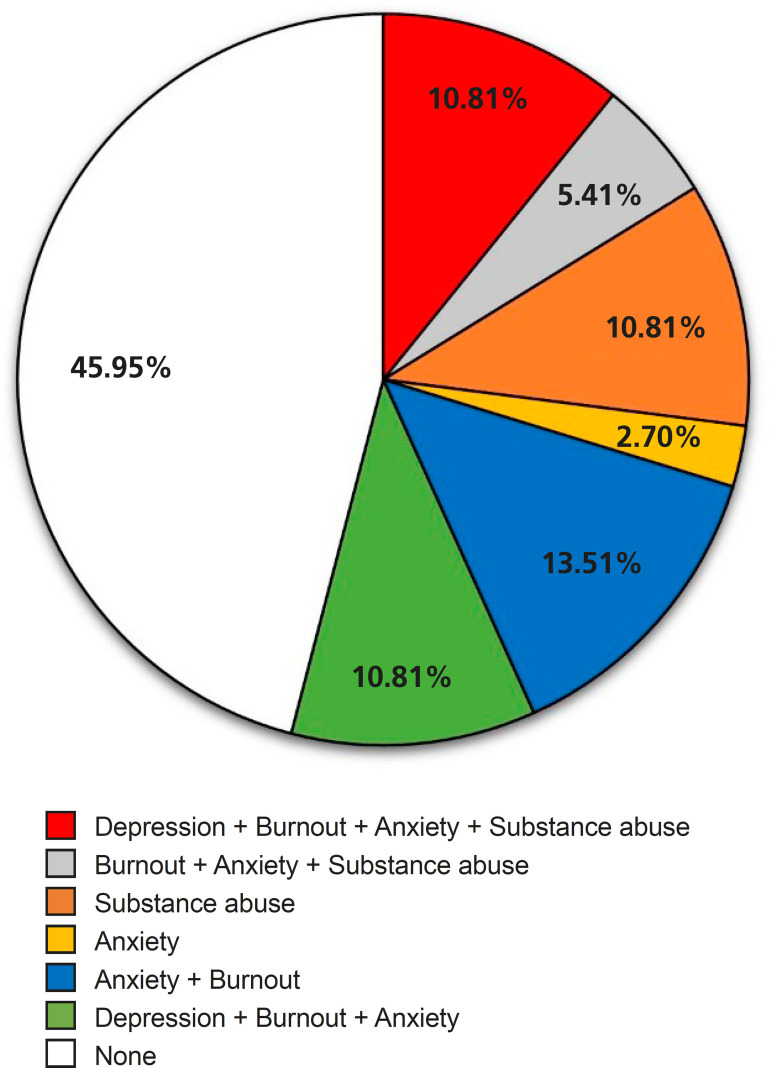
Presence of any change in the assessment of mental health.

Seventeen of the thirty-seven individuals studied (45.95%) did not present any disorder. Twenty research subjects (54.05%) had at least one disorder. Participants were grouped according to their responses to the questionnaire into one or more nosological groups -16 individuals (43.24%) were anxious, eight (21.62%) were depressed, 10 (27.03%) were drug or alcohol users, and 15 (40.54%) were in burnout (Figure 1). Therefore, 13.51% of the participants showed signs of anxiety and burnout, 5.41% were characterized with anxiety, substance abuse and burnout, the abuse of illicit drugs and alcohol was represented by four (10.81%) of the interviewees, no participant presented signs of anxiety and depression, burnout and anxiety were found in two (2.70%) cases, and four individuals (10.81%) had signs suggestive of all nosologies (Figure 2).

## DISCUSSION

The suffering of some of the surgeons could be pointed out by difficulties in relationships, alcohol and drug abuse, suicide, and some somatizations such as diarrhea, trismus, spinal problems, and difficulty in falling asleep or early awakening. These manifestations are part of the questionnaire used in this research and they were applied as an indication of mental illness. Diseases were included in groups in a simplified way, as an example: anxiety, depression, burnout, and drug use. Because of that, a validated form (the DSM-5(R)) was chosen to identify these behavioral changes among Brazilian cardiovascular surgeons. Several studies have demonstrated the relationship between surgical specialties and psychiatric diseases, although there are no validated studies that demonstrate this association with cardiovascular surgeons^[2,6]^.

Another study related the number of nights on call of surgeons with the occurrence of burnout. Working two or more nights on duty per week is associated with the onset of the disease in up to about 46% (*P*<0.0001)^[7]^. It is interesting to note that surgeons who work more than eighty hours a week obtained a higher rate of medical errors when compared to doctors who worked less than sixty hours per week (10.7% and 6.9%, respectively; *P*<0.001)^[7]^; in addition, the first group is twice as likely to attribute the error to emotional exhaustion (*P*=0.001). The specialties with more hours of work and night shifts in this study were traumatology, cardiovascular surgery, urology, and transplant^[7]^.

In a survey with 3,807 surgeons, 45% of respondents under the age of 60 years considered leaving their careers in the past two years. The three main reasons for this were excessive stress, high demand for work time, and shortage of personal time (*P*<0.0001), despite job satisfaction (80%)^[8]^. This study also analyzed job satisfaction with stress factors in cardiothoracic surgeons and orthopedists and demonstrated that the three most important stressors were responsibility in the operating room (z=3.42; *P*<0.001), balance in professional life, and workload compared to medical assistants^[8]^. Medical satisfaction is closely related to the sense of personal fulfillment, that is, it is a predictor of burnout syndrome.

According to the latest report by the World Health Organization for mental disorders, in Brazil, there is a prevalence rate of anxiety of 9.3% and it is 5.8% for depression^[3]^, while the prevalence rates expressed in our results were 21.62% and 27.03%, for anxiety and depression, respectively. In addition, our data showed a prevalence of 40.54% for burnout, against 32% in the general Brazilian population. Therefore, surgeons are more predisposed to have mental disorders than the general population, when comparing these studies with our results^[8]^.

Throughout their careers, many doctors are frustrated by the difficulty in reaching goals they had at the beginning of it. Remuneration and career development below expectations are factors that increase this frustration. At the same time, they will perceive, over time, the high professional demands and dedication necessary to exercise their profession. Despite the stressful profession, it seems that retirement is also a trigger for depression and other disorders such as burnout^[2]^.

Most surgeons are aware of disorders such as depression and anxiety. However, the manifestations of burnout, emotional exhaustion, depersonalization, and a lesser sense of personal fulfillment are poorly understood and may be associated with the high demands of the profession^[8]^.

With regard to drug abuse, a retrospective cohort showed that surgeons were significantly more likely to seek rehabilitation services after five years of follow-up (odds ratio [OR] 1.9; 95% confidence interval, 1.3-2.7; *P*=0.001), compared to clinical doctors^[9]^. The study also concluded that more surgeons had retired, given up the profession, had their medical records revoked, or died at the end of the follow-up, compared to non-surgeon doctors^[10]^. Another study related alcohol abuse by surgeons and the greater probability of medical errors over a three-month period (OR 1.45; *P*=0.001)^[5]^. According to a study, surgeons who showed symptoms of depression were more likely to make medical errors, compared to surgeons with negative screening for the disease (54.9% *vs*. 27.5%; *P*=0.0001)^[10]^.

The need for actions to create more organized work environments is evident. The organization of the operating room generates a better environment and less stress for surgeons. Establishing routines can minimize errors and generate a healthier place of work. In addition, avoiding long working hours, practicing physical activities, a healthy social life, and having hobbies to abstract a little from the professional activity can help to reduce anxiety and control moments of aggressiveness, decreasing the pressure and producing beneficial effects as well.

### Limitations

It is important to note that we distributed 120 questionnaires at the congress and only got 37 responses. This may be a sign of negligence or denial of the problem by surgeons and may have caused some bias in our study. Anyway, there are no specific studies on cardiovascular surgery and mental health, and we hope to alert about this problem. The interpretation of the questionnaires can be biased. The results represent the impression of the analyst psychiatrist. This psychiatrist did not personally interview the research subjects. He interpreted the questionnaires completed by the participants. In addition, qualitative studies, which study impressions, have limitations as demonstrated.

## CONCLUSION

The prevalence of mental illnesses such as anxiety, depression, drug abuse, and burnout was very frequent among cardiovascular surgeons in Brazil in 2019.

**Table:** 

Authors' roles & responsibilities	
EAVR	Substantial contributions to the conception of the work; and analysis of data for the work; drafting the work; final approval of the version to be published
FRA	Substantial contributions to the acquisition of data for the work; drafting the work; final approval of the version to be published
ACMN	Substantial contributions to the acquisition of data for the work; drafting the work; final approval of the version to be published
LLK	Substantial contributions to the acquisition of data for the work; drafting the work; final approval of the version to be published
BSMG	Substantial contributions to the acquisition of data for the work; drafting the work; final approval of the version to be published
LAP	Substantial contributions to the acquisition of data for the work; drafting the work; final approval of the version to be published
DRT	Substantial contributions to the acquisition of data for the work; drafting the work; final approval of the version to be published
ACABS	Substantial contributions to the acquisition of data for the work; drafting the work; final approval of the version to be published
GLM	Substantial contributions to the analysis of data for the work; final approval of the version to be published
